# Predatory bacteria can protect SKH-1 mice from a lethal plague challenge

**DOI:** 10.1038/s41598-019-43467-1

**Published:** 2019-05-10

**Authors:** James S. Findlay, Helen C. Flick-Smith, Emma Keyser, Ian A. Cooper, E. Diane Williamson, Petra C. F. Oyston

**Affiliations:** CBR Division, Dstl Porton Down, Salisbury, SP4 0JQ UK

**Keywords:** Antimicrobial resistance, Pathogens

## Abstract

With the rise of antimicrobial resistance, novel ways to treat bacterial infections are required and the use of predatory bacteria may be one such approach. *Bdellovibrio* species have been shown *in vitro* to predate on a wide range of other Gram-negative bacteria, including CDC category A/B pathogens such as *Yersinia pestis*. The data reported here show that treatment of SKH-1 mice with *Bdellovibrio bacteriovorus* HD100 provided significant protection from a lethal challenge of *Yersinia pestis* CO92. This is the first report of protection conferred by predation *in vivo* against a systemic pathogen challenge. However, this protective effect was not observed in a preliminary study with Balb/c mice. Therefore the effects of the predatory bacteria are complex and may be dependent on immune status/genetics of the host. Overall, predatory bacteria may have utility as a therapeutic modality but further work is required to understand the predator-host interaction.

## Introduction

Over recent decades, there has been a reduction in the rate of discovery of new antibiotics whilst there has also been an increase in resistance to existing antibiotics, a situation that has spawned the term “antibiotic apocalypse”^1^. Therefore there is a need to develop new therapies to treat bacterial infections. *Bdellovibrio* and like organisms (BALOs) are small, Gram-negative bacteria, found in both terrestrial and aquatic environments, that predate on other Gram-negative bacteria. *Bdellovibrio* enter the periplasmic space of the prey bacteria, replicate and then lyse the prey to release progeny predator^2^. Therefore one such approach to treating bacterial infections could be to use predatory bacteria such as *Bdellovibrio* as a ‘living’ antibiotic.

Recently, there has been increasing research evaluating predatory bacteria *in vitro* for their ability to kill a range of Gram-negative bacteria from diverse genera, including multi-drug resistant clinical isolates^3,4^. In turn this has led to research into the efficacy of *Bdellovibrio* species to treat bacterial infections *in vivo*, as well as evaluation of the safety profile of administration of predatory bacteria. As a Gram-negative organism, there would be concern about endotoxin having a detrimental effect on the host. Early studies showed that in addition to the degradation of macromolecular compounds of the prey to provide nutrition for the invading bacteria, *Bdellovibrio* recycled outer membrane proteins, lipid A, and fatty acids of the prey for integration into its own membrane system^5–9^. It has been demonstrated that the *Bdellovibrio* LPS is chemically different from the prey upon which the bacteria had fed: the lipid A has an unusual chemical structure which conferred low endotoxic activity in cytokine induction assays^10^. Initial work demonstrated that oral administration of predatory bacteria to young chicks was not associated with any adverse effects^11^. A subsequent study showed that inoculation of C57BL/6 mice with *Bdellovibrio* via the intranasal (i.n.) and intravenous (i.v.) routes resulted in an initial host response to the predator, and these pro-inflammatory cytokines (IL-1β, IL-6, IL-10, CXCL-1/KC, IFN-γ and TNF-α) returned to baseline levels after 18 hours^12^. Similar results were obtained in rats^13^.

Efficacy of predatory bacteria to control bacterial infections *in vivo* has been tested in a range of models. Injection into the hindbrain of zebrafish with *Bdellovibrio* increased survival of *Shigella*-infected zebrafish and the protective effect was the outcome of synergy between bacterial predation and host immunity^14^. Some efficacy has been reported in control of bacteria on surfaces: studies on *K. pneumoniae* in rats demonstrated that *Bdellovibrio* can reduce pathogen burden in lungs^15^. However, the predator was not able to control intravenously administered *Klebsiella* circulating in blood nor dissemination to the other organs, leading the researchers to suggest that “predatory bacteria may not be effective for treatment of acute blood infections”^13^. Similarly, in an experimentally induced infectious bovine keratoconjunctivitis model, treatment with *Bdellovibrio* was similarly ineffective, although the experimental model used produced lesions that did not mimic naturally occurring disease well^16^. Therefore, although *in vivo* studies demonstrated that the predatory bacteria had no obvious deleterious effects, efficacy *in vivo* has not been demonstrated convincingly. However, due to the pressing need for new approaches to combat infectious disease, interest remains high. We hypothesised that as the zebrafish model demonstrated a synergy between predation and host immunity, it may be that protective effects may be missed due to the model employed^14^. We wished to evaluate this hypothesis in a well-characterised systemic, acute disease model.

Predatory bacteria may have utility as a therapy to treat biological threat agents, including the pathogen *Yersinia pestis*, the causative agent of plague^17^. *Y. pestis* is a Gram-negative bacterium mainly infecting small mammals and is most commonly transmitted to humans by flea vectors to cause bubonic plague. Plague is endemic in a number of countries and is associated with seasonal outbreaks, such as the 2017 outbreak in Madagascar. Plague can be transmitted in infectious aerosols to cause pneumonic disease, with a case-fatality rate of up to 100% and antimicrobial resistant isolates have been identified^18,19^. Although the pathogen has an obligate intracellular phase during infection, *Y. pestis* is primarily an extracellular pathogen (as reviewed in^20^). As such, antibodies can be sufficient for protection following vaccination^21^. As *Bdellovibrio* can predate *Y. pestis in vitro*^17^, we considered that systemic plague may be a suitable challenge on which to test our hypothesis, especially as the mouse model has been extensively characterised. Therefore in this study, the ability of *B. bacteriovorus* HD100 to predate *Y. pestis in vivo* and potentially protect mice from a lethal challenge was investigated.

## Results

### *Bdellovibrio* can survive phagocytosis by murine macrophages *in vitro*

**Figure 1:**
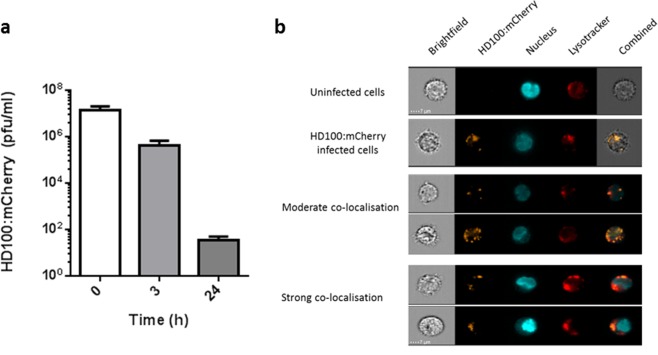
*B. bacteriovorus* HD100:mCherry can enter and persist in murine macrophages. (**a**) Survival of HD100:mCherry predator in J774A.1 cells over 24 h. (**b**) Representative images from the ImageStream analysis of uninfected J774A.1 cells or cells infected with HD100:mCherry showing co-localisation between the HD100:mCherry and Lysotracker Deep-Red 3 hours post-infection.

As *Y. pestis* has an intracellular phase during infection, we initially wished to determine whether *B. bacteriovorus* HD100 was able to survive in murine macrophages following phagocytosis. This would be important for identifying if the predator could target the intracellular *Y. pestis* prey, which persist in phagosomes, or would be limited to targeting extracellular prey. Using a mCherry-expressing strain of *B. bacteriovorus* HD100 (HD100:mCherry), an uptake assay was carried out on J774A.1 cells. No cell detachment or cytopathic effect was observed in infected versus uninfected macrophages. Whilst predator numbers reduced over time (approximately a three-log drop in viable predator by 24 hours), predator could still be isolated from J774A.1 cells suggesting that *B. bacteriovorus* HD100:mCherry could enter macrophages and survive following phagocytosis (Fig. 1a). To confirm that the predator was intracellular, J774.1 cells with *B. bacteriovorus* HD100:mCherry were infected and stained 3 hours post infection using Lysotracker Deep-Red (which only emits a strong signal under acidic conditions such as within organelles) and analysed via ImageStream. A mCherry signal could be detected and upon analysis, nearly all the signal (94 ± 0.49%) was co-localised with the Lysotracker signal, indicating that the vast majority of *B. bacteriovorus* HD100 in these cells 3 hours post infection were intracellular and appeared to be within the phagosome (Fig. 1b). Therefore *B. bacteriovorus* HD100:mCherry appeared to be able to survive intracellularly for sufficient time relevant to the treatment of an intracellular infection.

### Monitoring *Bdellovibrioin vivo*

**Figure 2:**
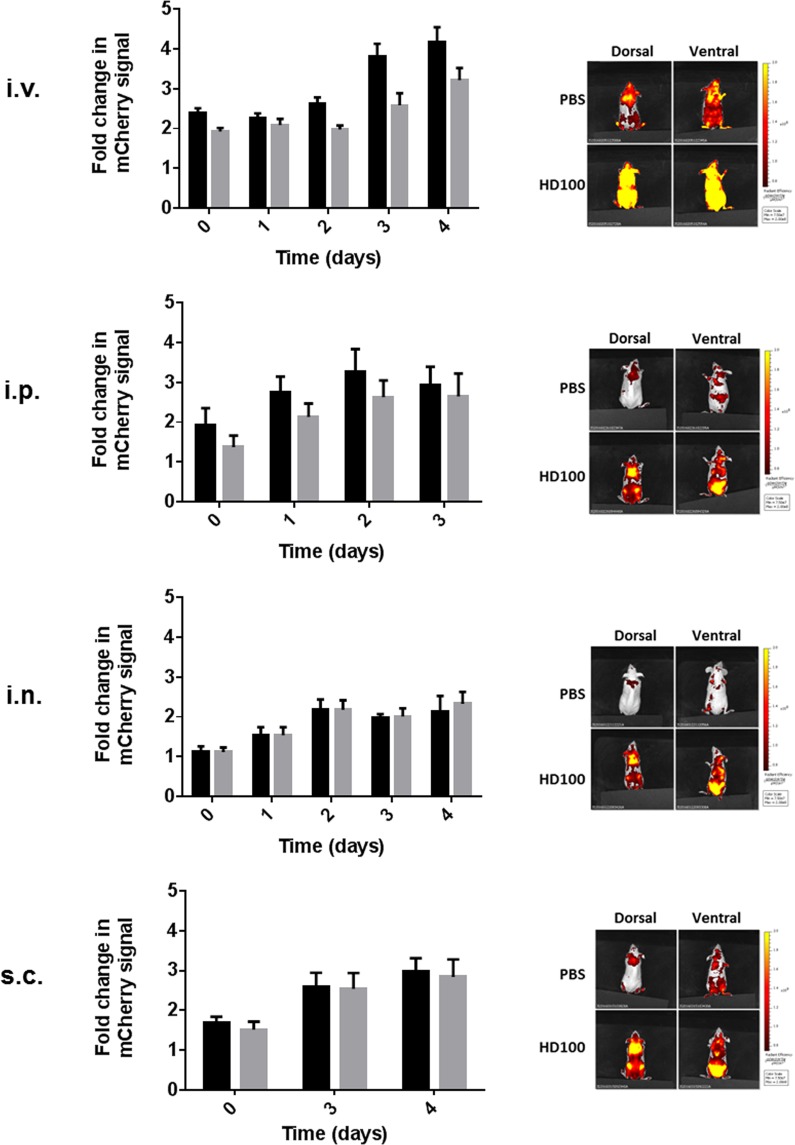
mCherry signal can be detected *in vivo* over 96 h after a single dose of *B. bacteriovorus* HD100:mCherry. Total body fluorescence (570/620 nm) of mice (groups of 3) dosed via the i.v., i.p., i.n., or s.c. routes with either a single dose of HD100:mCherry or PBS imaged daily for up to 5 days, except for s.c. groups which were not imaged until Day 3 due to technical issues. Data presented as fold change in mCherry signal in HD100:mCherry dosed mice compared to the signal in PBS-dosed mice on the dorsal side (black bars) and the ventral side (grey bars). Included for reference is a representative mouse from PBS- or HD100:mCherry-dosed groups imaged on the final day (all final day images are included in Supplementary Fig. S1).

**Figure 3:**
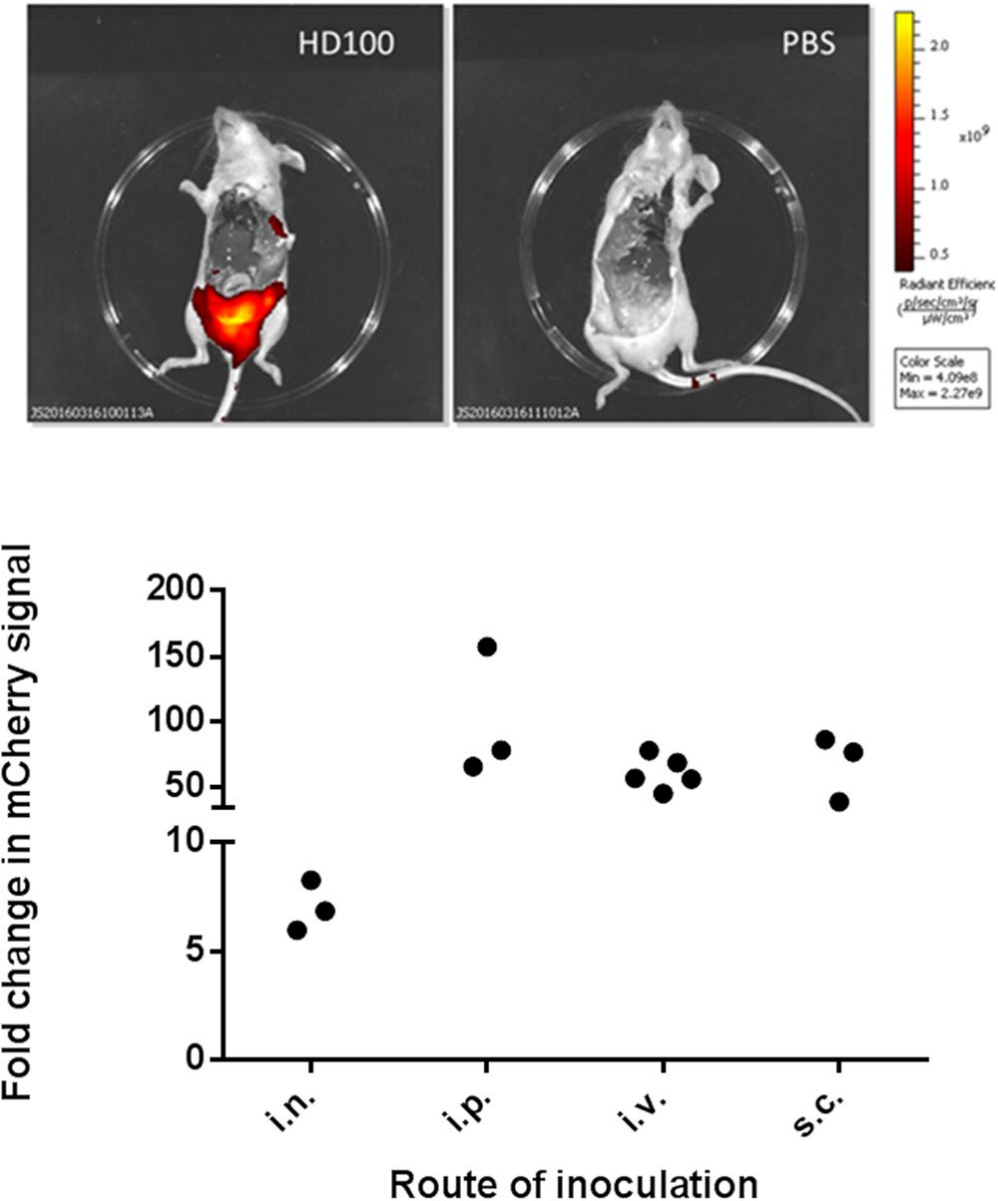
SKH-1 mice dosed with *B. bacteriovorus* HD100:mCherry had a strong signal associated with the inguinal adipose tissue. Top - representative picture of a mouse dosed with HD100:mCherry or PBS showing signal located with inguinal adipose tissue. Bottom - levels of mCherry signal detected in adipose tissue of mice dose with HD100:mCherry via four different routes. Data presented as fold change in mCherry signal in adipose tissue isolated from HD100:mCherry-dosed mice compared to the signal in control adipose tissue isolated from PBS-dosed mice.

**Figure 4:**
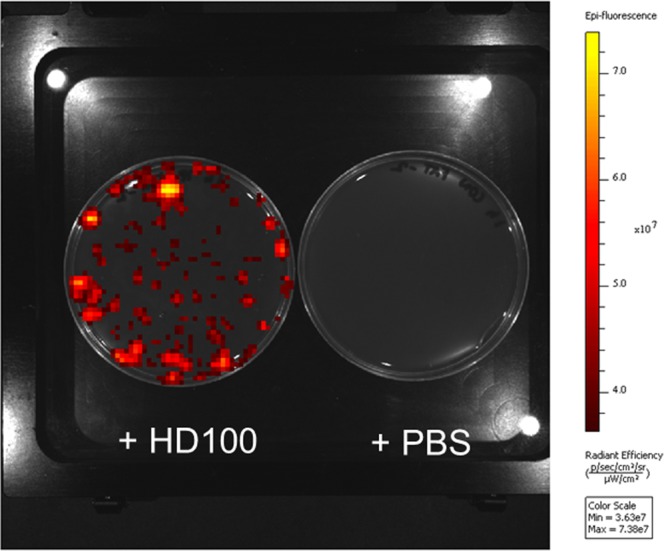
Predator isolated from fat from a mouse dosed with *B. bacteriovorus* HD100:mCherry (left) was enumerated by plaque assay and imaged using the IVIS. As a comparison, adipose tissue from mice dosed with PBS (right) was also processed and imaged.

To determine persistence and tropism of *B. bacteriovorus* HD100 *in vivo*, mice were inoculated with a single dose of *B. bacteriovorus* HD100:mCherry and the spread of the mCherry signal was followed with *in vivo* imaging. To reduce issues caused by auto-fluorescence of fur, SKH-1 mice (which are naturally hairless) were used. The predator was inoculated via four different routes at the following doses: i.v., 1.1 × 10^7^ pfu/mouse; intraperitoneal (i.p.), 5 × 10^8^ pfu/mouse; i.n., 4.8 × 10^8^ pfu/mouse; and sub-cutaneous (s.c.), 3.1 × 10^8^ pfu/mouse. The animals were imaged from 1 hour after inoculation (Day 0) for up to 5 days, and an increasing mCherry signal was observed over this time for all routes of inoculation (Fig. 2). At the end of the study, mice were culled and autopsied and the strongest mCherry signal was located in the adipose tissues of mice inoculated with *B. bacteriovorus* HD100:mCherry (Fig. 3). In an attempt to confirm the signal was related to presence of predator, a range of techniques were employed including PCR, fluorescent microscopy and plaque assays to varying levels of success. During all the imaging studies, no viable predator could be enumerated via plaque assay from excised adipose tissue. Viable predator was isolated on one occasion from one adipose tissue sample from a parallel study (Fig. 4). Therefore, whilst there have been issues with recovery and detection of bacteria, the imaging data and downstream analysis suggests that *B. bacteriovorus* HD100 does persist *in vivo* and may have an adipose tissue tropism.

### *B. bacteriovorus* HD100 protects SKH-1 mice from lethal challenge of systemic plague

**Figure 5:**
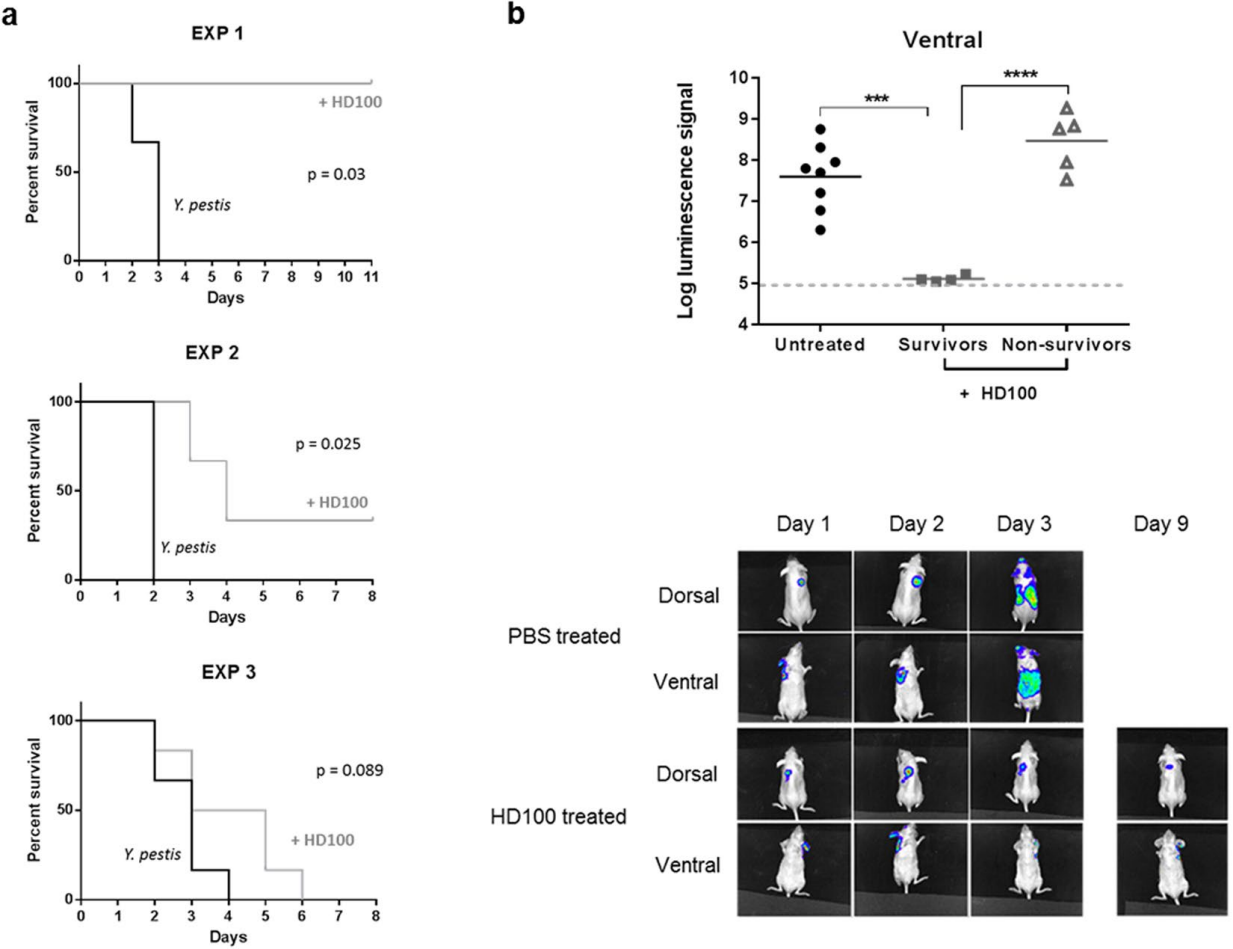
*B. bacteriovorus *HD100:mCherry protects SKH-1 mice from a lethal challenge of *Y. pestis*. (**a**) Survival curves of mice infected with *Y. pestis* CO92 pGEN-luxCDABE and treated with PBS (black lines) or HD100:mCherry (grey lines). Significance was evaluated using a log-rank test. (**b**) Bio-luminescence data from mice upon culling which were untreated, survivors or non-survivors. The grey dotted line is the level of background signal. The images are from a representative mouse from the PBS-treated (top) or HD100:mCherry-treated (bottom) groups from Experiment 1. *** = p < 0.001, **** = p < 0.0001, based on a two tailed, un-paired t-test.

For bioimaging, we took advantage of a luciferase-expressing strain of plague, *Y. pestis* CO92 pGEN-luxCDABE^22^. To determine if the predatory bacteria *B. bacteriovorus* HD100 were able to protect mice from a lethal challenge of *Y. pestis*, SKH-1 were pre-treated with *B. bacteriovorus* HD100:mCherry via the i.p. route and then infected with 1116 cfu (average across three experiments) of *Y. pestis* CO92 pGEN-luxCDABE via the s.c. route. Starting 24 hours after infection, mice were treated with predator daily and the spread of *Y. pestis* was followed with *in vivo* imaging. Treatment of mice with *B. bacteriovorus* HD100:mCherry resulted in a significant protection (p < 0.05) in two of the three studies (Fig. 5a). When analysed using a stratified log rank test, the predatory bacteria provided a highly significant (p < 0.001) protective effect from a lethal challenge of *Y. pestis* CO92 pGEN-luxCDABE. Mice which survived had no clinical signs and had a significantly (p < 0.0001) lower level of luciferase signal on the ventral side, suggesting that *B. bacteriovorus* HD100 was controlling the spread of *Y. pestis* from the site of infection (Fig. 5b). Subsequent enumeration of viable bacteria in the spleen at the end of the study identified three of the four mice which survived had no detectable bacteria and <10 cfu were detected in the other mouse, indicating that *B. bacteriovorus* HD100 provides protection by controlling spread of *Y. pestis* and achieving clearance of organs.

**Figure 6:**
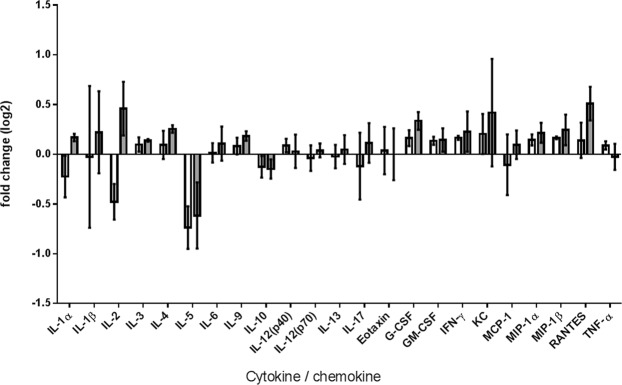
No significant difference between host responses in mice infected with a lethal dose of *Y. pestis* CO92 pGEN-luxCDABE in the presence or absence of *B. bacteriovorus* HD100:mCherry. Level of induction of cytokines/chemokines in SKH-1 mice infected with *Y. pestis* in the presence (grey bars) or absence (white bars) of *B. bacteriovorus* HD100, compared to the levels found in uninfected mice dosed with PBS, expressed as fold change (log_2_).

**Figure 7:**
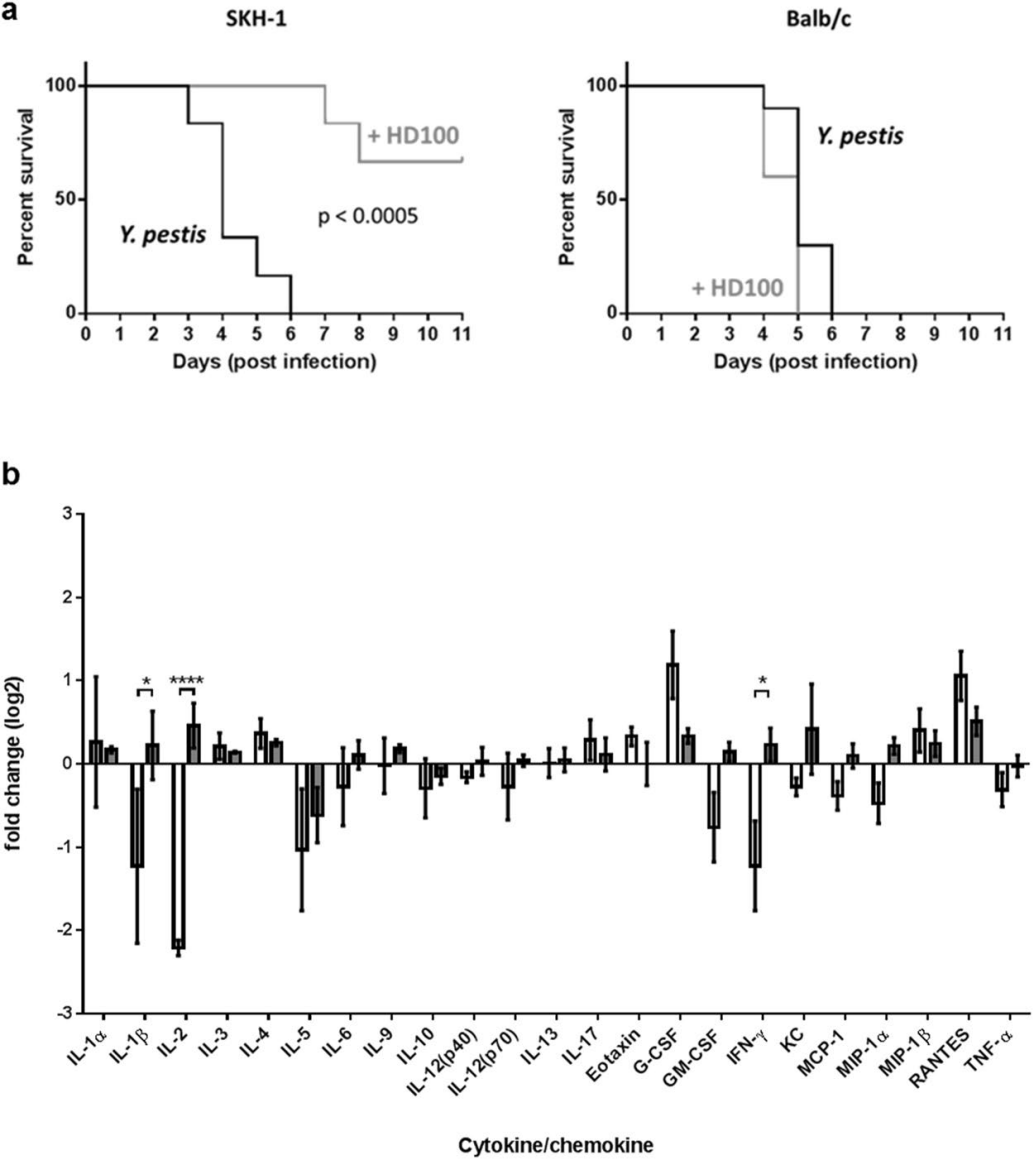
*B. bacteriovorus* HD100:mCherry is protective in SKH-1 mice but not in Balb/c mice. (**a**) Survival curves of SKH-1 (left) or Balb/c (right) mice infected with *Y. pestis* CO92 pGEN-luxCDABE and treated with PBS (black lines) or HD100:mCherry (grey lines). (**b**) Levels of induction of cytokines/chemokines of *Y. pestis* infected Balb/c and SKH-1 mice treated with HD100. Data is displayed as fold change (log_2_) compared to base line levels (uninfected mice dosed with PBS) for HD100:mCherry-treated Balb/c mice (white bars) and HD100:mCherry-treated SKH-1 mice (grey bars). * = p < 0.05, **** = p < 0.0001, based on a two-way ANOVA with Sidak’s correction for multiple comparisons.

Previous data in zebrafish showed that the host’s response could enhance the activity of the predatory bacteria^14^. To determine if the presence of HD100 was affecting the host’s response, the levels of cytokines in SKH-1 mice infected with *Y. pestis* in the presence or absence of *B. bacteriovorus* HD100:mCherry were compared after 24 h (Fig. 6). Comparison of the response in the presence or absence of *B. bacteriovorus* HD100:mCherry did not identify any cytokines which were significantly different between SKH-1 mice receiving HD100:mCherry or PBS. Whilst SKH-1 are a useful tool for imaging, they are less widely used than other strains of mice such as Balb/c. Therefore a small, preliminary study was carried out comparing the outcome in SKH-1 and Balb/c mice. Interestingly, no protection was seen in Balb/c mice, suggesting that there may be strain specific effects of the predatory bacteria (Fig. 7a). The cytokine responses in *Y. pestis-*infected Balb/c mice receiving HD100 were compared to those of the *Y. pestis*-infected SKH-1 mice receiving HD100 (Fig. 7b). Treatment with HD100 (in *Y. pestis*-infected mice) resulted in a differential response, with three cytokines (IL-1β, IL-2 and IFN-γ) being significantly higher in infected SKH-1 mice compared to Balb/c mice, suggesting that the differential protection seen may be linked to host response to the predator in the context of prey.

## Discussion

This study has demonstrated that *B. bacteriovorus* HD100 provided significant protection from a systemic challenge of *Y. pestis* in SKH-1 mice. This is the first report of protection against systemic challenge in a mammalian model. However, it must be reported that in a small, preliminary study in Balb/c mice, *B. bacteriovorus* HD100 did not show protection. Further studies will be required to validate this preliminary observation.

In this study, there appeared to be an adipose tissue tropism of *Bdellovibrio in vivo*. This has not previously been observed, but probably because previous studies in mice^12^ used culture of prey bacteria or qPCR of organ extracts as the output, rather than whole body imaging, as used here. The interaction of predatory and prey bacteria in the zebrafish model^14^ would also not have indicated this phenomenon due to different anatomy between piscean and mammalian hosts. Adipose tissue has been implicated as a reservoir of infection for viruses including HIV^23^ and mouse adenovirus type 1^24^, bacterial pathogens, including *Mycobacterium tuberculosis*, which residues inside adipocytes of fat tissue^25^ and parasites such as *Trypanosoma cruzi*^26^. Furthermore dendritic cells, which are pivotal in initiating immune responses, exist in adipose tissue which they use as an energy source^27^. Whilst we detected a fluorescent signal associated with adipose tissue in all cases (and this was specific to mCherry and not general fluoresence), we were only able to isolate predator once from adipose tissue. This could reflect that standard isolation process of predator may be insufficient for adipose tissue or that the predator may not rely on prey-dependent growth. However it cannot be ruled out that some of the detected signal was due to accumulation of the mCherry protein after lysing of the predator in serum. Therefore, further work is required to optimise isolation of viable predator and identify within which specific cell type the predator resides, potentially using qPCR as in other reported studies^28^.

Plague presents as two distinct phases of disease: an extended “pre-inflammatory” phase during which bacterial replication occurs in the absence of discernible disease symptoms or inflammatory responses; and a subsequent “pro-inflammatory” phase characterized by the onset of symptoms, dramatic increases in cytokines, neutrophils, and inflammatory lung pathology. Maintenance of the extended pre-inflammatory state, despite inflammasome activation, allows for unimpeded bacterial replication^29^. In our studies, the presence of *B. bacteriovorus* HD100 alone did not have a deleterious effect in either mouse strain, which supports the finding that dosing i.n. or i.v. with BALOs resulted in a modest but short-lived inflammatory response in mice with no impact on survival^12^. Similarly, human phagocytic cells produced lower pro-inflammatory cytokine levels when infected with *Bdellovibrio* versus two members of the *Enterobacteriaceae*^30^. *B. bacteriovorus* HD100 provided significant protection from *Y. pestis* in SKH-1 mice. It is not possible to define the mechanism by which *B. bacteriovorus* HD100 was protective, for example if it involves active predation, triggering the host to respond to the infection or by blocking entry into phagocytic cells. The preliminary data suggests that there may be a strain specific effect as similar protection was not seen in Balb/c mice (Fig. 7). Whilst the Balb/c mouse is well characterised, less is known about SKH-1 mice. The SKH-1 mouse, being hairless, was originally used for dermatologic and wound healing research (reviewed by^31^). The strain is immunocompetent, outbred, unpigmented and genetically uncharacterised. The immunobiology of various hairless strains has been evaluated^32^ and although some differences are reported with responses in fully furred mouse strains, these mice can reject skin allografts and form antibodies normally. In addition, humoral and cellular immunity of the SKH1 mouse line determined that blood counts, immunoglobulin levels, and CD4^+^ and CD8^+^ T cells were comparable between SKH1 and the C57BL/6 strain^33^. Well-established paradigm states simplistically that C57BL/6 mice show Th1 biased immune responses, while Balb/c mice have more balanced responses, with a tendency towards Th2. This is reflected in differences in the ability to generate protective responses upon immunisation and subsequent challenge for some pathogens eg *Francisella tularensis*^34^, or in differences between inbred mouse strains in the level of resistance to eg *Pseudomonas aeruginosa* infection^35^. Both C57BL/6 and Balb/c mice are susceptible to *Y. pestis*, whereas *Mus spretus* SEG mice can resist *Y. pestis* and develop an immune response characterized by a strong recruitment of M1 macrophages^36^. This resistance correlated with stronger bactericidal activity with higher nitric oxide production, a more pro-inflammatory polarized cytokine response, and a higher resistance to *Y. pestis*-induced apoptosis^36^. In addition, other studies have shown that the dampening of the immune response in *Y. pestis* infected mice with corticosteroids improves bacterial clearance, immunopathology and survival in mice receiving antibody therapy^37^. In our studies, analysis of the host response to a single dose of *B. bacteriovorus* HD100 in *Y. pestis*-infected mice showed that there was a differential host response, with three pro-inflammatory cytokines being higher in SKH-1 mice than Balb/c mice. Therefore given the role that the immune response can have to the outcome of plague, the increase in pro-inflammatory cytokines suggest that SKH-1 were able to control *Y. pestis* proliferation *in vivo* by ablation of the pre-inflammatory stage of plague. Alternatively, SKH-1 mice were able to control the harmful inflammatory response of late infection whilst allowing predation to work synergistically with the immune system to remove the pathogen. Further work is required to delineate the relative contribution of predation or immune response to outcome.

The finding that *B. bacteriovorus *HD100 protected SKH-1 mice from lethal challenge with *Y. pestis* expands the potential opportunties for predatory bacteria to be used therapeutically. This is consistent with a recent observation that repeated dosing with *Bdellovibrio* reduced colonisation of the lung in pneumonic plague, although impact on survival was not reported^38^. In this study, the therapy was delivered pre-exposure and then given once a day. Further studies are needed to determine the effect of post-exposure therapy, as well as if more than one daily dose could be given. However, Balb/c mice data suggests that the effects of predatory bacteria *in vivo* may be complex and need to be elucidated if predatory bacteria are to be used as a therapeutic. Similarly the interaction between the predatory bacteria and the host immune system, and how this affects disease progression, needs to be elucidated to determine the relative contribution of predation vs immune response to protection. Therefore whilst this is the first report of protection against systemic challenge in a mammalian model, further work is needed to evaluate the utility of predatory bacteria as a therapeutic.

## Methods

### Buffers and media

Ca/HEPES buffer: 5.49 g/l HEPES free acid, 0.284 g/l calcium chloride dehydrate, pH 7.6. YPSC overlay agar: 0.25 g/l magnesium sulphate, 0.5 g/l sodium acetate, 1 g/l Bacto peptone, 1 g/l yeast extract, 6 g/l agar, pH 7.6; adjust to 0.25 g/l anhydrous calcium chloride after autoclaving. YPSC ‘hard’ agar: same as overlay agar except 10 g/l agar added.

### Growth and preparation of predatory B. bacteriovorus HD100

*B. bacteriovorus* HD100:mCherry, a strain which a mCherry fluorescence protein tag on the Bd0064 protein, was grown as previously described^14,39^. Briefly, *B. bacteriovorus* stocks were prepared by culture on stationary phase *E. coli* S17-1 prey suspended in Ca/HEPES buffer (5.49 g/l HEPES free acid, 0.284 g/l calcium chloride dehydrate, pH 7.6). *E. coli* cells were grown for 16 h in L broth at 37 °C. To grow predator, a ratio of 3:1 prey: predator was used and cultures incubated at 28 °C for 24 h at 180 rpm. After two 24 h cultures, the resultant lysate was used to set up cultures for the experiments. For assays, cultures ranging from 10–250 ml were set up and incubated for 40 h. To purify and concentrate predator, the lysate was passed through a 0.45 µm filter and pelleted and washed twice with PBS by centrifugation. After the final wash, the cells were re-suspended in a sterile PBS at a 100x concentrated stock. Predator input was subsequently enumerated by serial dilution and enumeration of plaques in YPSC overlay agar (containing *E. coli* S17-1) which was on top of YPSC hard agar as previously described^39^. For heat-killed predator, prepared predator was incubated at 105 °C for 10 mins, followed by cooling and enumerated to confirm that no viable predator could be detected.

### Intracellular persistence of B. bacteriovorus HD100 in murine macrophages

The J774.1 murine macrophage cell line was seeded in wells of a 24-well plate at 5 × 10^5^ cells/well in DMEM supplemented with 10% foetal calf serum (FCS) and 2 mM glutamine. Cells were incubated overnight, and in the morning the media was removed and cells washed with PBS. Predator was added in L-15 media supplemented with 2% FCS and the cells/predator incubated for 2.5 h in a 37 °C, non-CO_2_ incubator. After this incubation time, gentamicin was added to the cells at 50 μg/ml gentamicin (final concentration) and the cells incubated for a further 0.5 h. The input was removed and fresh media containing gentamicin. At set times, the cell medium was removed, cells washed twice with PBS and then the cells lysed by addition of dH_2_O. Viable predator was then enumerated by overlay assays. Prior to infection and at each sampling time, monolayers were visually examined. For ImageStream analysis, rather than cells being lysed by addition of dH_2_O, cells were scraped into PBS, stained with 40 nM Lysotracker Deep-Red and Hoescht 33342 dye and run on the ImageStream X. Over 7,000 events were collected and analysed using the following strategy: identification of single cells, identification of these with mCherry signal and then within this subset, the level of co-localisation of the masks set for the mCherry signal and the Deep-Red signal. Across all three experiments, the average number of cells collected was 7521 cells, and of these, 92% of the cells across all three experiments were positive for mCherry signal.

### Growth and preparation of Y. pestis

*Y. pestis CO92 pGEN-luxCDABE*^22^ was grown for ~16 h in BAB broth supplemented with 50 µg/ml ampicillin at 28 °C, 180 rpm in a shaking incubator in an biosafety level 3 laboratory. This culture was diluted 1 in 10 in fresh BAB broth supplemented with 50 µg/ml ampicillin and grown at 28 °C, 180 rpm in a shaking incubator until OD_590_ was 0.6 (an OD which is approximately 1 × 10^8^ cfu/ml). The culture was then diluted to the appropriate concentration in PBS and used for the challenge. The challenge dose was subsequently enumerated after challenge by determining viable colonies on BAB plates (supplemented with haemin and 50 µg/ml ampicillin) and incubated at 28 °C for 3–4 days. For enumeration of *Y. pestis* from mice, organs were removed, homogenised in PBS through a cell sieve and plated onto BAB agar containing 50 µg/ml ampicillin.

### Animal studies

All procedures involving animals were conducted in strict accordance with the Animals (Scientific Procedures) Act 1986 under project licence 30-3166. This project licence was approved following an ethical review by Dstl’s Animal Welfare and Ethical Review Body. Female Balb/c mice, and male or female immune-competent but hairless SKH-1 mice (both supplied by Charles River Laboratories) aged 6–8 weeks old, were used in these studies. On arrival, mice were randomly assigned to groups (group size defined in figure legends) and allowed to acclimatise to biosafety level 3 isolator conditions for a minimum of 5 days. Prior to challenge, mice were given approximately 1 × 10^8^ pfu of mcherry expressing *Bdellovibrio* in a total volume of 50 µl by the i.p. route. Mice were subsequently challenged approximately 2 h later with ~1000 pfu of *Y. pestis* CO92 pGEN-luxCDABE given in a volume of 100 µl by the s.c. route on the dorsal side. Mice were administered a single dose (50 µl i.p.) of the freshly-prepared predator daily, until the end of the studies. Control mice were treated identically but given PBS instead of predator. For imaging studies, mice were anaesthetised using ketamine-medetomidine (0.78 mg Ketaset, Fort Dodge Animal Health Ltd, Southampton, and 0.015 mg Domitor, Elanco, Basingstoke, given in a total volume of 200 µl i.p.) and once unconscious, imaged using an IVIS Spectrum (Caliper, Perkin Elmer, USA) and images captured and analysed using the Living Image (ver. 4.5) software to detect bioluminescence or fluorescence signal, using the built in auto-exposure function of the software. To reduce background fluorescence mice were fed an alfalfa-free diet (AIN93M, LabDiet) from 7 days before the start of the studies. After imaging, mice were recovered using Atipamezole (Antisedan, Janssen Animal Health, High Wycombe, 0.05 mg in 133 µl i.p.) for imaging on consecutive days. Whilst under anaesthetic mice were kept in a warming box and closely observed until fully recovered. Mice were weighed daily and checked at least twice daily for clinical signs of disease. Upon reaching predetermined humane end points mice were culled by cervical dislocation.

### Cytokine assay

Blood samples were obtained from anaesthetised mice (groups of 3 mice) via cardiac puncture. The blood was incubated at room temperature to allow the blood to clot and the samples centrifuged at 11,000 rpm for 15 minutes. The serum was removed and stored in −80 °C and assayed at a later date using Bio-Rad Luminex 23-plex, group 1 pro-inflammatory cytokine assay kit. Briefly, samples were thawed, processed according to manufacturer’s instructions (using neat serum) and analysed on a Bio-Rad Plex 200 system.

### Statistics

Survival curves were compared using the log-rank test on Graphpad v6. Stratified log-rank tests were performed on SPSS. The luciferase data for adipose tissue was analysed using a two tailed, un-paired t-test of the log transformed data (t = 9.261 df = 7). Cytokine log_2_ data was analysed by a two-way ANOVA with Sidak’s correction for multiple comparisons on Graphpad v6 (df = 92). All error bars shown are standard error of the mean.

## Supplementary information

Supplementary Figure 1

## Data Availability

All relevant data are within the paper or the Supplementary Fig. online.
