# Supplementary material for: Predatory bacteria can protect SKH-1 mice from a lethal plague challenge
Source: Sci Rep. 2019 May 10;9:7225. doi: 10.1038/s41598-019-43467-1 (PMC6510791; doi:10.1038/s41598-019-43467-1)
Supplement: Supplementary file 1 — Supplementary Figure 1 [file 41598_2019_43467_MOESM1_ESM.docx]

**Predatory bacteria can protect SKH-1 mice from a lethal plague challenge**

James S. Findlay, Helen C. Flick-Smith, Emma Keyser, Ian A. Cooper, E. Diane Williamson and Petra C.F. Oyston


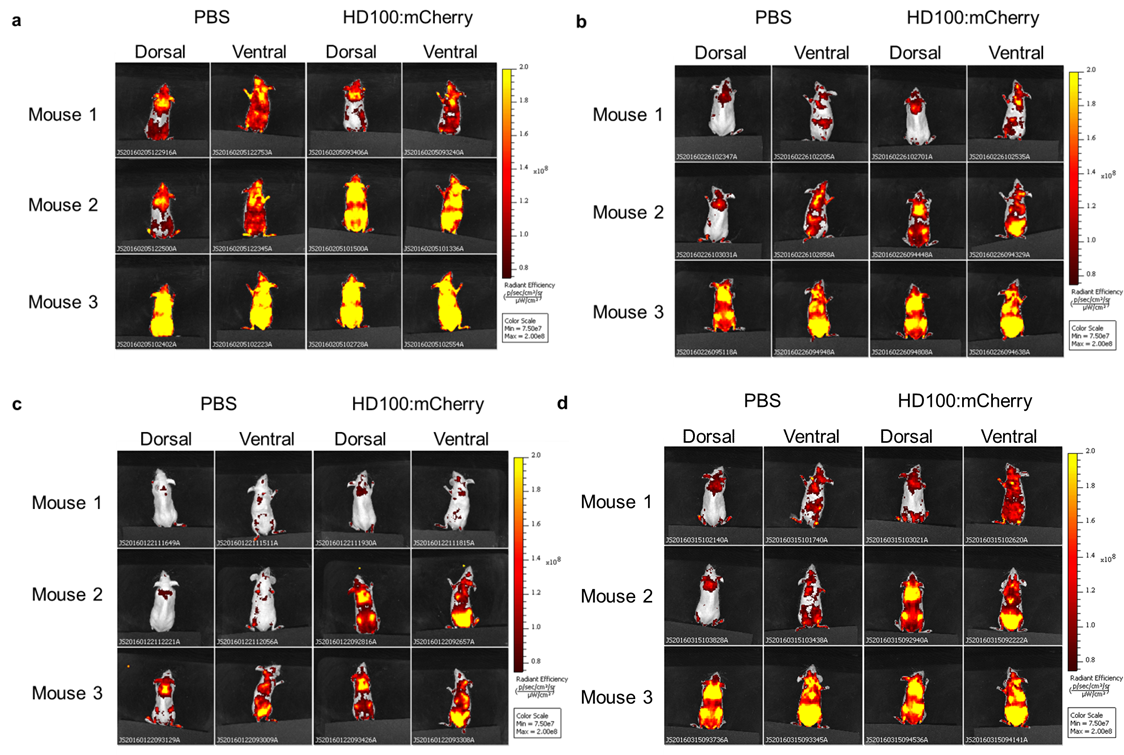


Supplementary Figure S1 - IVIS images of all SKH-1 mice given a single dose of HD100:mCherry. The predator was inoculated on Day 0 via the a) i.v. b) i.p. c) i.n. or d) s.c routes. Images were taken on the final day of the studies (using 570 nm excitation/620 nm emission). All images are on the same fluorescence scale to allow direct comparison.
